# Advancing School Behavioral Health Implementation: A Critical Review Using ISF 2.0

**DOI:** 10.3390/bs16091663

**Published:** 2026-09-16

**Authors:** Nicole S. Litvitskiy, Kristen Figas, Margaret D. Hall, Maya Resnick, Abraham Wandersman, Paul Flaspohler

**Affiliations:** 1 Department of Psychology, Miami University, Oxford, OH 45056, USA; hallm3@miamioh.edu (M.D.H.); resnicme@miamioh.edu (M.R.); flaspopd@miamioh.edu (P.F.); 2 Department of Psychology, University of South Carolina, Columbia, SC 29208, USA; kfigas@email.sc.edu; 3 Wandersman Center at Fors Marsh, Columbia, SC 29201, USA; wanderah@mailbox.sc.edu

**Keywords:** implementation, school behavioral health, readiness

## Abstract

As youth behavioral health needs in the United States continue to grow, schools are increasingly important settings for prevention and service delivery. Yet evidence-based practices (EBPs) are not consistently implemented or sustained at scale. In this article, we present a critical narrative review of dissemination and implementation efforts in school behavioral health (SBH) through the lens of the Interactive Systems Framework for Dissemination and Implementation 2.0 (ISF 2.0). We reviewed and synthesized relevant SBH and implementation science literature to identify promising practices, persistent challenges, and gaps in the field, and then applied the ISF 2.0 as an analytic lens to examine how these issues operate across the systems involved in SBH implementation. Findings suggest that misalignment in goals, strategies, roles, and communication across systems can impede the delivery of EBPs to schools in ways that are feasible, responsive to local needs, and sustainable. Drawing on these findings, we developed ISF 2.0–guided questions and applied them to three prominent SBH implementation initiatives: the National Center for School Mental Health (NCSMH), the Center on Positive Behavioral Interventions and Supports (Center on PBIS), and the South Carolina School Behavioral Health Academy (SBHA). Selected based on their national or regional prominence, accessibility of information, and implementation activities, these illustrative cases demonstrate how the ISF 2.0 can be used to identify cross-system strengths, gaps, and opportunities for improvement. Although this review is narrative rather than systematic and the cases are illustrative rather than exhaustive, the analysis highlights the importance of stronger bidirectional communication and greater attention to how knowledge is generated, translated, adapted, and used in partnership with stakeholders. The ISF 2.0 provides a promising framework for understanding the complex systemic factors shaping SBH implementation and for strengthening alignment among the systems that generate knowledge, support implementation, and deliver services. Greater cross-system alignment should help schools implement and sustain effective behavioral health practices that are responsive to community needs and improve youth well-being.

## 1. Introduction

Rising youth mental health concerns in the U.S. indicate that it is more imperative than ever to ensure quality delivery of effective interventions like school behavioral health (SBH) programs. SBH initiatives aim to support students’ behavioral health through universal prevention, early intervention, positive behavioral supports, and systems-level approaches (Weist et al., 2022a). Through delivery in youth’s everyday environment (Hoffmann et al., 2022; Manning, 2009), such efforts are key to the promotion of positive behaviors and prevention or reduction in behavioral concerns (Weist et al., 2020) and addressing the gap of rising unmet needs (Leeb et al., 2024) for increasing youth mental health concerns (Woolf, 2025). However, implementation challenges often impede efforts to achieve desired outcomes, with ongoing barriers and gaps pointing to a need for improved understanding of SBH challenges and development of innovative solutions (Richter et al., 2022; Schäfer et al., 2025). While many implementation frameworks and models have been developed to synthesize determinants and effective practices for dissemination and implementation, their sustained application to real-world contexts remains limited (Balis et al., 2025). Therefore, it is essential to examine current practices through novel approaches to promote effective implementation. In this article, we propose that the Interactive Systems Framework for Dissemination and Implementation 2.0 (ISF 2.0; Wandersman et al., 2024) is uniquely designed to provide a potential roadmap for diagnosing breakdowns in implementation and proposing solutions for effective SBH programming.

### 1.1. School Behavioral Health Implementation

SBH involves the intentional integration of behavioral and mental health supports into school’s multi-tiered systems of support (MTSS), a prevention and intervention framework designed to match students to support appropriate for meeting their needs (Barrett et al., 2013). This tailored approach includes prevention and intervention at universal (Tier 1), targeted (Tier 2), and individual (Tier 3) levels (Evans et al., 2017). Evidence consistently supports the positive impact of integrating intervention and prevention programming into school systems (Hoover & Bostic, 2021; Weist et al., 2021, 2022b), with the potential to support positive youth development in a cost-efficient manner (McCrone, 2025). However, significant barriers remain to the uptake and delivery of quality programming. Effective implementation hinges on schools having the knowledge and skills to implement SBH practices (capacity), as well as recognizing the value of SBH and perceiving it to be worth the effort (motivation; Flaspohler et al., 2008). Weaknesses in capacity and/or motivation hinder SBH delivery, resulting in a failure to achieve desired outcomes at scale (Price et al., 2024; Splett et al., 2022).

### 1.2. Implementation Frameworks for SBH

The need to enhance SBH implementation requires a comprehensive understanding of the processes and determinants that guide implementation. Numerous implementation frameworks offer insight into the factors that may impact successful delivery of school-based services (Albers et al., 2017). Frameworks often differ in their structure and distinguishing features, each lending unique elements to the understanding of implementation efforts (Hanson et al., 2016). For example, the Consolidated Framework for Implementation Research (CFIR) integrates components from multiple models and theories to catalog determinants–barriers and facilitators–of implementation (Damschroder et al., 2022). Other models, such as the Exploration Preparation Implementation and Sustainment (EPIS) framework, detail stages of implementation (Hanson et al., 2016). Another process-focused model, the Getting to Outcomes (GTO) framework, highlights an approach to comprehensive implementation that guides stakeholders through identifying the problem, establishing goals, selecting an evidence-based program that “fits,” building capacity within the organization, developing a plan, and engaging in continuous evaluation and quality improvement to ensure sustainability (Wandersman et al., 2000). Collectively, these frameworks and models address the know-do gap—that is, how to improve the use of evidence in real-world practice settings (Beidas et al., 2025). While important, these efforts are persistently constrained by what we know. The complexity of large-scale community-based implementation efforts requires not just understanding the implementation process and contextual factors that impact its success, but also understanding how knowledge is produced, translated, and disseminated (Graham et al., 2006; Wandersman et al., 2008). This requires a different type of framework, one that identifies the systems involved across the entire continuum linking knowledge production to use.

The Interactive Systems Framework for Dissemination and Implementation 2.0 (ISF 2.0; Wandersman et al., 2024) expands the lens beyond local delivery to include how evidence is distilled, packaged, and disseminated via three key systems involved in translating research, providing implementation support, and delivering community-based services. This framework emphasizes the need for systems to demonstrate readiness—a product of motivation, general capacities, and innovation-specific capacities—to effectively engage in efforts to disseminate and implement prevention and intervention programming (Scaccia et al., 2015). With systems situated in a broader context (society), the ISF 2.0 details the “how” of moving from the development of evidence-based interventions to successful implementation efforts by delivery systems (e.g., schools). Importantly, the ISF 2.0 is intended to be a “living skeleton,” supported by the understandings from implementation science (i.e., other models, theories, and frameworks) and ongoing expansion based on novel developments (Wandersman et al., 2024, p. 4). In the context of SBH implementation, this systems-level framework allows for a more comprehensive understanding of the efforts and interactions of systems involved in providing school-based support.

### 1.3. Current Review

Barriers to SBH implementation impede efforts to support youth’s social, emotional and behavioral needs. Evidence supporting the effectiveness of SBH, including numerous empirically supported programs and treatments, are insufficient for actualizing change at scale in school-based settings. Significant efforts have been made to bridge the gap between research and practice via development of resources and tools to disseminate evidence-based knowledge to community-based practitioners and trainers. To address ongoing challenges within these efforts, the overarching purpose of the present study is to conceptually explore the feasibility and utility of applying a systems-level perspective (i.e., ISF 2.0) to SBH implementation efforts. Therefore, the study has three aims:

(1) Provide a broad overview of the status of the field of school behavioral health, specifically identifying best practices and limitations of its implementation;
(2) Critically evaluate this synthesized literature through the lens of the ISF 2.0 in order to determine what works best across the systems involved in SBH implementation;
(3) Develop an initial set of guiding questions and demonstrate the potential of their application to evaluating three current SBH implementation support efforts.

To accomplish these aims, we first conduct a critical narrative review of SBH implementation literature to consider current SBH practices in the context of the ISF 2.0. Based on this review, we use the ISF 2.0 as a guiding framework to develop a preliminary analytical lens through which to identify gaps and inform solutions across three examples of current SBH initiatives. In sum, we use the ISF 2.0 to illustrate the necessity and value of a systems-level perspective for strengthening current approaches to the youth mental health crisis.

## 2. Method

**Figure 1:**
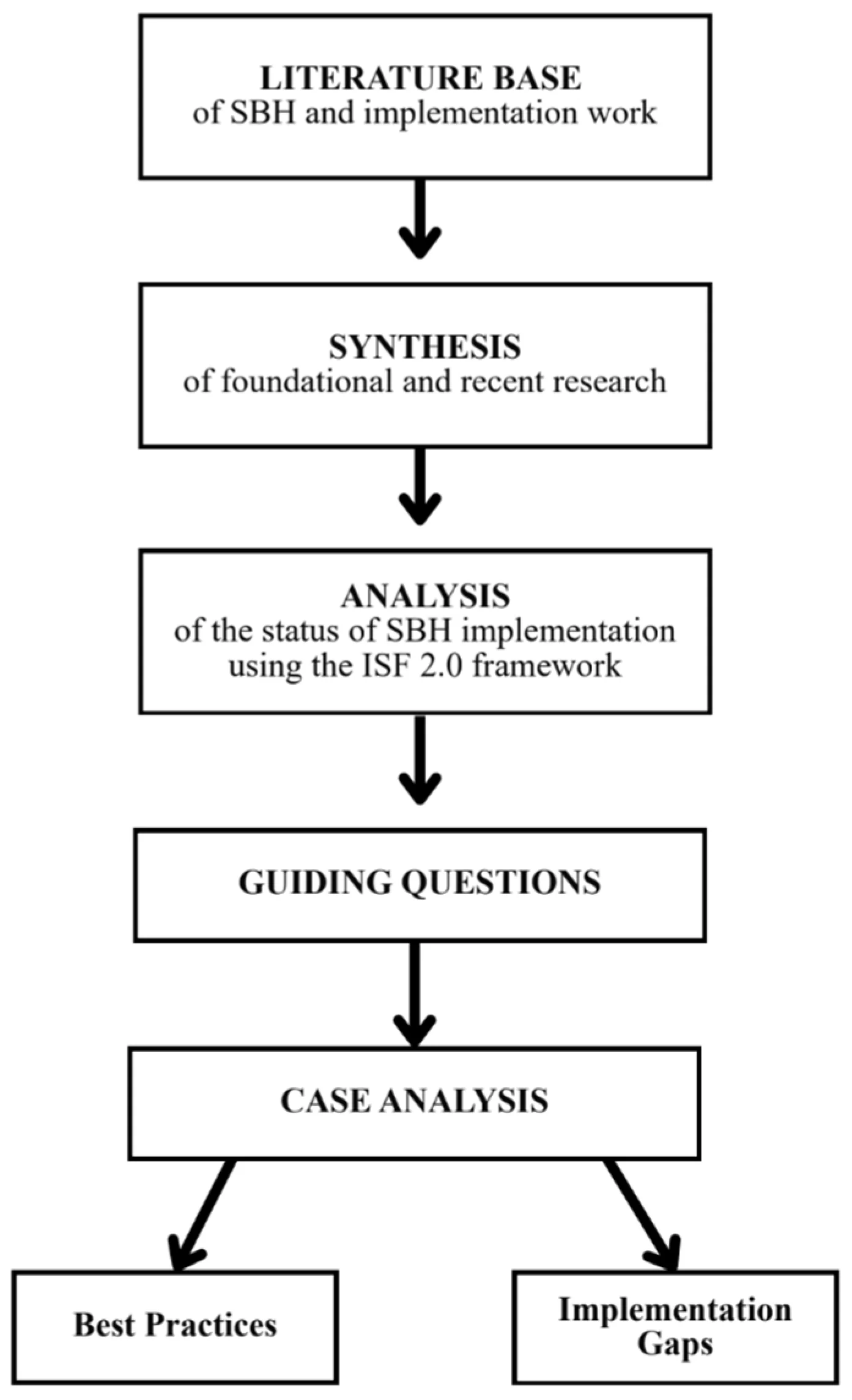
Visual Representation of Methodological Process.

A critical narrative literature review was conducted to explore existing efforts in SBH implementation. Narrative reviews provide a broad, descriptive synthesis of the current status on a topic (Gregory & Denniss, 2018). A critical review is a subtype of narrative review that applies an interpretative lens, grounded in theory, to explicate gaps that can only be exposed by synthesizing diverse studies (Sukhera, 2022). For the present study, a critical narrative review was conducted to integrate SBH and implementation science literature to suggest best practices and highlight continued gaps that must be addressed. This approach is consistent with the purpose of such reviews in presenting an argument based on existing research (B. N. Green et al., 2006). The critical review of the SBH and implementation science literature in this study is informed and interpreted through the lens of the ISF 2.0 framework. See Figure 1 for a visual illustration of our methodology.

### 2.1. Selection Process

#### 2.1.1. Literature Review

Sources for this narrative review were identified primarily through authors’ prior research, database searches, and citation tracking. Literature gathered from previous work were selected with the same inclusion criteria as noted below. These articles were primarily papers that are well-known in the SBH and/or implementation science fields and may be considered foundational and/or influential literature. This process was supplemented through a literature search using multiple databases and search engines, such as Google Scholar, PubMed, and PsycINFO. A combination of keywords and search terms, using the Boolean operators “AND” and “OR,” was used to identify articles addressing the following concept domains: school settings; behavioral and mental health; prevention and intervention programming; implementation science; implementation determinants and outcomes; implementation processes, strategies, and supports. Representative search terms included *school-based*, *school behavioral health*, *school mental health*, *multitiered systems of support*, prevention program*, intervention*, evidence-based practice*, *implementation science*, implementation strateg*, *implementation support*, *adoption*, *fidelity*, *sustainment*, *dissemination*, determinant*, barrier*, facilitator*, *technical assistance*, *capacity building*, and *program evaluation*.

Article titles and abstracts were reviewed first to assess for relevance before full-text articles were examined to confirm suitability. In addition, citation searching across all initially identified sources was done and screened in the same process. Articles were selected for inclusion based on content relevance to the primary focus of the article (i.e., school behavioral health programming; implementation). Articles were excluded if the focus was solely on schools outside of the U.S. or were not published in English. Implementation literature from outside the U.S. was included if the content was deemed relevant. There was a primary focus on articles published within the last 15 years (2011–2026) in order to better characterize the current field of SBH implementation; however, select older articles were included as determined to be especially important or relevant. The selection of articles presented below is non-exhaustive but were chosen to highlight areas of implementation best practices and barriers to effective SBH and provide a basis to point to gaps in current efforts.

#### 2.1.2. Case Examples

Three cases were selected to illustrate real SBH implementation efforts among professional organizations. School behavioral health focused organizations were chosen based on criteria such as national recognition (well known within the broader field), accessibility of relevant information (e.g., published papers on efforts, extensive website information, authors’ own connections to the organization), and clear implementation efforts across multiple ISF 2.0 systems (collaborations across systems or efforts focused on more than one area). Information about these cases were supplemented by data from the organizations’ websites, research publications and reports, and insight from authors’ own experiences with the organizations. The National Center for School Mental Health (NCSMH) was selected as an illustrative example for its well-established reputation within the field working across national, state, and district levels. The Center on Positive Behavioral Interventions and Support (PBIS) was chosen based on its work connecting supports to schools themselves. The Center on PBIS is a federally funded center operating on national, state, and district levels, and PBIS is highly recognized by and used within school settings. Finally, the South Carolina School Behavioral Health Academy (SBHA) was selected as a representative of a state-level partnership and for its focus on integrating behavioral health into existing school frameworks of support (e.g., multi-tiered system of supports, or MTSS). The second author has direct experience with the SBHA and was able to more readily provide information about its functioning for an illustrative case example. In sum, out of many existing implementation support efforts, these three cases were chosen due to their familiarity for readers and their particular relevance to illustrating different aspects of best practices and gaps in implementation efforts.

### 2.2. Procedure

First, a broad synthesis of current SBH implementation efforts was completed based on the literature search, including effectiveness of programs and implementation strategies and barriers. Then, the reviewed SBH implementation literature was interpreted using the ISF 2.0 framework by organizing the reviewed literature across the ISF 2.0 systems. Based on this literature review, key concepts of SBH implementation within the ISF 2.0 framework were identified and distilled into guiding questions for the case analysis. Finally, three selected case examples were evaluated in the context of the ISF 2.0 systems using some of these questions. Gaps in implementation efforts for each case example were further analyzed and critiqued using the ISF 2.0. Consistent with critical review attributes (e.g., Saunders & Rojon, 2011; Sukhera, 2022), the results (1) describe relevant and significant research on SBH implementation, (2) evaluate selected articles through the lens of the existing ISF 2.0 framework, and (3) use representative case examples from the field to exemplify key features from the literature.

## 3. Synthesis of Current SBH Implementation Efforts

Significant efforts have been made to develop successful SBH programming and evaluate associated outcomes. A substantial body of literature demonstrates the effectiveness of specific SBH practices across universal prevention/promotion programs (e.g., Durlak et al., 2011; Osher et al., 2016; Sklad et al., 2012; Taylor et al., 2017), targeted intervention for students displaying early concerns (e.g., Bruhn et al., 2014; Cho Blair et al., 2021), and intensive intervention for students experiencing significant emotional and behavioral challenges (e.g., Goh & Bambara, 2012; Mychailyszyn et al., 2012). Many of these programs and interventions have been translated into manuals and guidance materials to facilitate their use in practice settings. Overall, there is strong evidence of the benefit of these programs, but research indicates mixed findings about implementation efforts. Literature, reviewed below, highlights significant implementation challenges and offers strategies for improving SBH implementation.

### 3.1. Implementation Barriers

Despite the extensive library of evidence-based programming for prevention and intervention, in “real-world” practice, SBH programming often fails to achieve desired outcomes, such as improved academics and reduced mental health concerns. Several factors within the implementation process, such as access to training and the influence of organizational climate, may impact outcomes (March et al., 2022; Richter et al., 2022). For instance, strong implementation fidelity (i.e., delivering an intervention as intended and with high quality; Durlak & DuPre, 2008) relies on school personnel’s investment in a program and ability to manage the tasks related to its implementation (Moir, 2018). However, schools often lack the necessary knowledge and resources to maintain the capacity to implement these programs (Evans et al., 2013; Owens et al., 2014). Without ongoing collaboration between researchers or program developers and school personnel, the delivery of SBH programming can deviate extensively from the intended approach (Melde et al., 2006). Difficulties in reliable delivery of programming impact the evidence base, as methodological concerns within SBH research often mirror challenges in delivering and evaluating programs in school settings (Bartlett et al., 2017).

A significant challenge to implementation lies in the methodological concerns within SBH research. Barriers to conducting research in school settings (e.g., logistics of program delivery and data collection, need for parental involvement, and limitations of self-report data) impede efforts to translate on-the-ground experiences into evidence (Bartlett et al., 2017). As a result, the same reviews and meta-analyses that demonstrate the benefits of SBH also note the extensive methodological concerns among the existing research that evaluates school-based prevention and intervention programming. Specifically, heterogeneity of results and incomplete methodological reporting—including limited information on study context, sample characteristics, and implementation outcomes (e.g., fidelity)—stifle efforts to synthesize the evidence base (e.g., Durlak et al., 2022; Bennett et al., 2015; Bruhn et al., 2014; Grant et al., 2025; Krach et al., 2025; Lee et al., 2026; Mychailyszyn et al., 2012). Studies are also often characterized by high attrition rates and small sample sizes (Litvitskiy et al., 2026). Poor fidelity and methodological concerns can hamper the transfer and use of evidence in the practice space (Flaspohler et al., 2012; Khan et al., 2024). Without robust data from diverse school contexts, researchers are limited in their ability to guide schools in selecting and implementing SBH programming. Taken together, SBH literature indicates significant concerns with practical implementation as a result of barriers in the delivery and evaluation of SBH programming.

### 3.2. Best Practice Strategies

Despite persistent challenges to SBH implementation, research has identified best-practice strategies for effective implementation. Implementation support has long been identified as a key ingredient of successful SBH implementation (Sugai & Horner, 2006). Scholars have defined numerous strategies for supporting implementation in school contexts, such as ongoing training and consultation, monitoring implementation progress, and providing incentives (Cook et al., 2019). Existing reviews document the effectiveness of a variety of these strategies (Baffsky et al., 2023; Ennis et al., 2020; Konstantinidou et al., 2023; Merle et al., 2022; Solomon et al., 2012; Stormont et al., 2015).

Strategic partnerships between implementation support providers and school-based delivery agents are important for building schools’ motivation (e.g., commitment, perceived benefit) and capacity (e.g., school culture, staff capacity) for SBH delivery (Splett et al., 2022; Wandersman et al., 2008, 2024). These initiatives are accomplished through assembling multiple implementation strategies that engage school-based expertise, provide training and ongoing support to key staff, and promote collaborative efforts to change (Bohnenkamp et al., 2024; Oakes et al., 2014; Price et al., 2024). In particular, communal approaches that focus on strengthening partnerships and leveraging shared expertise through learning collaboratives have demonstrated promising results (Connors et al., 2020; Heatly et al., 2023; Pearrow et al., 2021). In general, strong program delivery benefits from collaborative approaches that engage school leaders and staff (Baffsky et al., 2023) and build buy-in for a program and its implementation (Moir, 2018).

### 3.3. Limitations in Existing Literature

Although the literature has extensively examined barriers to effective SBH implementation and strategies for addressing them, several important gaps remain. For example, while the importance of motivation and general capacity is acknowledged in the literature (e.g., Herlitz et al., 2020; Langley et al., 2010; March et al., 2022), relatively little is known about how to effectively develop these readiness components in the context of SBH. Additionally, whereas substantial research examines implementation support for SBH interventions (e.g., Baffsky et al., 2023; Merle et al., 2022; Stormont et al., 2015), comparatively little research has examined ways of supporting the foundational elements of SBH, such as teaming (e.g., Wolk et al., 2019), universal screening (e.g., Brann et al., 2021), and data-based decision making (e.g., Newton et al., 2011). Thus, there remain gaps in understanding how to support the implementation of key SBH components.

## 4. Analysis of SBH Implementation Efforts Through the ISF 2.0 Lens

Despite the expanding literature on evidence-based programming and implementation strategies, important questions remain about how these approaches can be coordinated across the systems involved in SBH implementation (Lyon & Bruns, 2019). Researchers develop strategies to support implementation, but there is limited evidence that these supports have been developed in collaboration to best address a school’s needs (Baffsky et al., 2023). This lack of communication between the various systems involved in SBH contributes to ongoing failures to support schools in delivering effective programs that meet community needs and promote positive youth well-being. Gaps in understanding and delivery underscore the need for guidance—for researchers, implementation support personnel, and school systems—on how to engage with one another to develop, implement, and evaluate programs that work (Lyon & Bruns, 2019). In this article, we consider how the ISF 2.0, an implementation framework that uniquely addresses the crucial interactions between systems, offers guidance on bridging such gaps.

### 4.1. Interpretative Lens: ISF 2.0

The Interactive Systems Framework for Dissemination and Implementation (ISF; Wandersman et al., 2008) was first developed to bridge the gap between research and practice by capturing the numerous perspectives and interest-holders involved in prevention and intervention efforts. Prior to the ISF, models of dissemination and implementation (e.g., from the Institute of Medicine, 1994) tended to focus on either the source (i.e., researcher or innovation developer) or the user of an innovation. These models failed to consider the bidirectional flow of knowledge across these perspectives (Wandersman et al., 2008). As a result, the ISF outlined three key systems that must function together to disseminate and implement effective programming: (1) delivery, (2) support, and (3) synthesis and translation.

These three systems are responsible for an ongoing and iterative cycle of synthesizing research, translating it into usable practices, and providing implementation support for the delivery of SBH services in schools. The synthesis and translation system is responsible for distilling research evidence for the other systems; the support system assists the delivery system in implementation; and the delivery system is tasked with carrying out the innovation. Importantly, knowledge and understanding must flow across these systems. For instance, while the synthesis and translation system is predominantly responsible for identifying and communicating evidence-based innovations to the support and delivery systems, the delivery system is a key part of outcome evaluation through the cyclical sharing of results back to the synthesis and translation system to further integrate into the evidence base. In this way, each system is instrumental in advancing the fields of implementation, prevention, and intervention.

**Figure 2:**
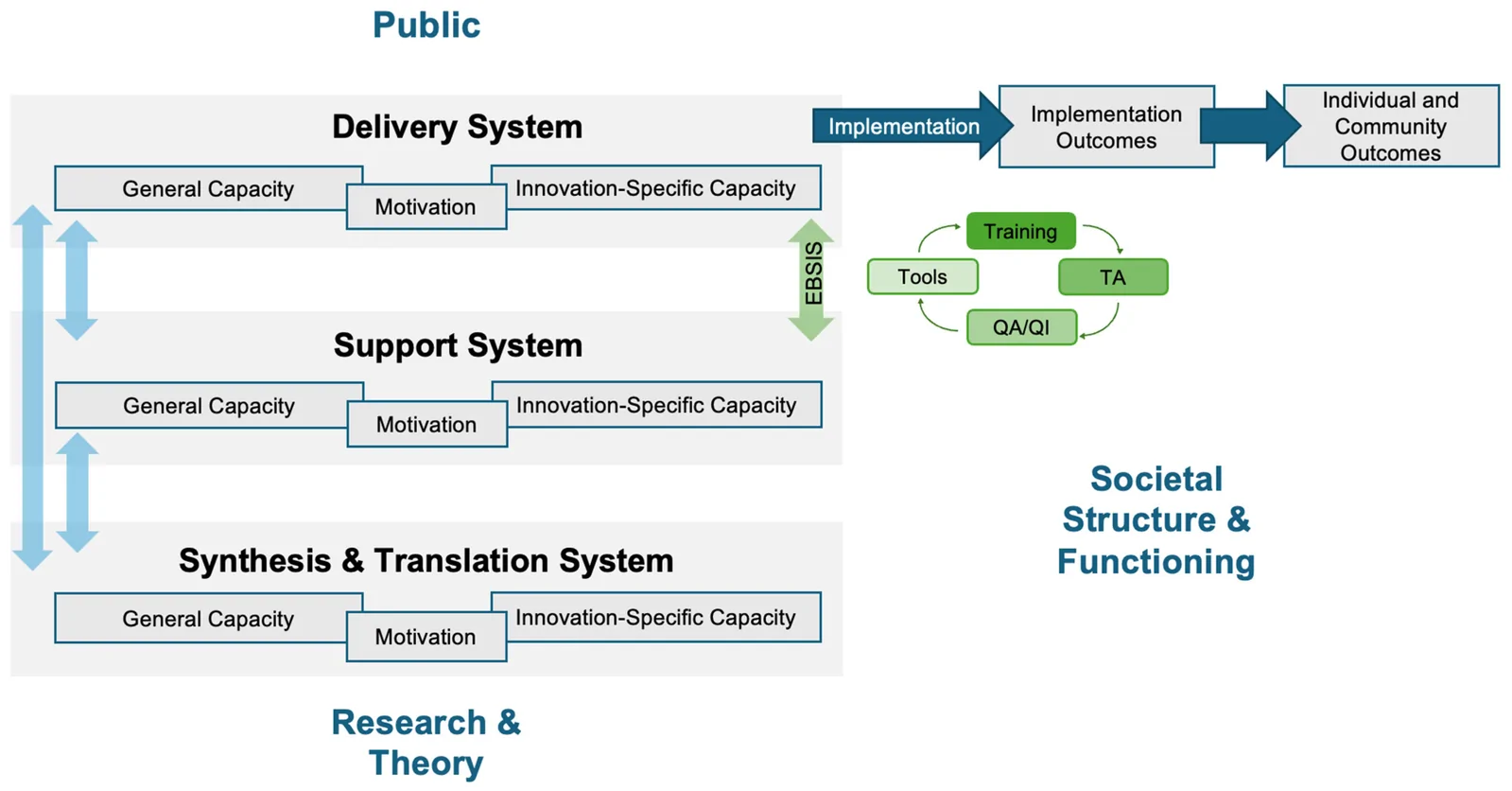
Interactive Systems Framework for Dissemination and Implementation 2.0. Note. This figure was adapted with permission from (Wandersman et al., 2008, 2024).

Since its inception, the ISF has been expanded to better capture the nuances of dissemination and implementation. The ISF 2.0 (see Figure 2) reflects the dynamic embeddedness of the three systems within society, emphasizes the importance of implementation and program outcomes, and introduces the idea of readiness to implement an innovation (Wandersman et al., 2024). The ISF 2.0 demonstrates that the three systems not only exist within a broader climate but actively interact with the public (those outside the systems who have a role in what the systems aim to achieve) and other societal systems (culture and climate, existing research and practice, funding, and policies and laws). Additionally, as depicted in Figure 2, the implementation arrow was added to demonstrate the goal of the three coordinated systems in producing and evaluating implementation outcomes (the degree to which the innovation is implemented with quality) and desired recipient outcomes (impact of the innovation itself on the targeted population). While each system is critical, the ISF 2.0 proposes all systems must function as co-creators of knowledge to achieve and maintain successful implementation outcomes. Finally, the ISF 2.0 reconceptualizes capacity building as just one piece that is necessary to the work of each system. Systems must have the readiness, which is a product of motivation, general capacities, and innovation-specific capacities, to effectively engage in efforts to disseminate and implement prevention and intervention programming. Compared to the original, the ISF 2.0 better captures the broader context (i.e., public, society), the importance of outcome evaluation, and system-specific motivation and capacities. These additions offer a novel perspective that may be used to drive future successful dissemination and implementation work in schools.

### 4.2. Interpretation of SBH Implementation Through ISF 2.0

From a research perspective, we delve into the ISF 2.0 systems, examining how relevant SBH literature on implementation and readiness fits within each ISF 2.0 system. Table 1 summarizes key concepts from SBH implementation research to ground our understanding of what works. Using this research-backed knowledge, the following sections explore the concept of SBH readiness (motivation, innovation-specific capacity, and general capacity) within each ISF 2.0 system. We provide a critical lens on how each system can engage in effective implementation practices, including active interaction between the systems. Then, we offer a conceptual guide to using the framework for diagnosis and problem-solving within the SBH implementation process.

#### 4.2.1. Delivery System

Delivery systems are the backbone of implementation. This system encompasses the environment in which the program is implemented and the people and institutions who facilitate and are impacted by the program. A motivated SBH delivery system is one in which its members (e.g., leaders, educators, practitioners) believe in the importance and benefit of program delivery. SBH must be communicated as a priority by leadership and valued by school staff, families, and students. This requires that SBH programming be aligned with other initiatives and “fit” the context of the school setting (Merrill et al., 2025). Programming must be feasible, sustainable, and acceptable to the individuals in the school (Herlitz et al., 2020; March et al., 2022). Delivery systems must also be able to collect and meaningfully communicate outcome data to school/district staff and leaders (Cook et al., 2019). The ability to effectively deliver SBH requires support and commitment (motivation) from multiple levels of leadership, belief in its benefits, and efforts to pilot and evaluate programming.

Delivery systems must also have the capacity to provide and evaluate SBH programs. This includes a culture of continuous improvement and reflection that respects and supports the views of staff and students (Cook et al., 2019). Leadership must demonstrate active engagement in the process, in the form of allocating resources, training staff, and demanding data-based decision-making. Doing so requires leaders to be knowledgeable about the implementation and quality improvement process and staff to be able to provide ongoing support for SBH implementation. Delivery personnel must clearly understand and communicate expectations around implementation, which hinges on adequate time, space, funding, and personnel allocation to SBH components. Beyond these components of general capacity, delivery systems must also be equipped with an understanding of core SBH practices and personnel should have clarity on their role. SBH delivery often benefits from teams established at multiple levels (e.g., district, school, for individual students) that collaborate to advance implementation efforts. Importantly, delivery systems cannot effectively achieve integrated SBH implementation without a supportive climate and champions who continue advancing the work.

Successful community-based participatory research (CBPR) has consistently demonstrated the benefit of partnerships to increasing the delivery systems’ motivation and capacity (Boothroyd et al., 2017; Collins et al., 2018; Salimi et al., 2012). An approach like CBPR is necessary for continued evaluation as well. In particular, a focus on empowerment evaluation highlights the importance of collaborating with delivery systems for the measurement of fidelity and outcome effectiveness (Fetterman et al., 2014; Fetterman & Wandersman, 2005). Partnerships with delivery systems prior to the start of implementation help to build the delivery system’s readiness at motivation and capacity levels as well as engaging them in sustainable practices through evaluations (Merrill et al., 2025; Schmittdiel et al., 2010). Overall, implementation with school delivery systems must be collaborative and incorporate a determination of the school’s readiness across systems (i.e., full school, classroom-level, etc.).

#### 4.2.2. Support System

The support system helps delivery system providers deliver evidence-based practices through structured professional learning and implementation support, often in the form of training and technical assistance centers (TTACs; Wandersman & Scheier, 2024). A motivated support system is tasked with providing implementation support that aligns with the needs and preferences of the delivery system. A training and technical assistance (TTA) “ecosystem” can allow for coordination among TTA providers from across different settings (Ward et al., 2024). This broad unification of TTA efforts from funders, researchers, and providers is important for a more collaborative and aligned TTA that is better suited to support the delivery system (Bumbarger et al., 2024). Evaluation of the effectiveness of support provision is also necessary to ensure the support system meets the needs of the delivery system (Wandersman & Scheier, 2024). In order to accomplish evaluation, support systems must be committed to continuous improvement through monitoring of the quality of implementation support (Bumbarger et al., 2024). Support providers require appropriate expertise in both SBH content and implementation processes. It is also critical that implementation support be considered an ongoing process and not simply a standalone training at the start of SBH delivery. This requires adequate funding and resources for sustained implementation support, as well as the integration of feedback throughout. Thus, support providers need to demonstrate fluency in SBH, best practices in training and technical assistance, and evaluation and quality improvement. As in the delivery system, leaders must advocate for provision of high-quality support and build strong, trusting relationships with delivery system partners.

The support system is key to communicating the value and importance of both SBH itself and implementation support for its delivery. Implementation support should be a priority that both support providers and delivery providers are committed to. Such support must be provided in a way that is understandable, feasible, accessible, and able to be received and used by the delivery system (e.g., Collier-Meek et al., 2016, 2017). The External Implementation Support Theory of Change has outlined 10 core practice components, which can be beneficial to apply to school settings. These practices include (1) building collaborative relationships, (2) reinforcing leadership self-regulation of effective implementation practice, (3) assessing implementation capacity, performance, and progress, (4) facilitating collaborative agreements about implementation goals, (5) teaching implementation science best practices to leaders, (6) facilitating the development of implementation capacity, (7) facilitating teams’ application of skills and resources in their context, (8) coaching leaders and teams, (9) facilitating collective learning and adaptive problem solving, and (10) transitioning out of intensive support (Aldridge et al., 2023). These mechanisms of change are a starting point for TTA researchers and practitioners to develop, measure, and select evidence-based strategies for enhancing implementation support.

#### 4.2.3. Synthesis and Translation System

The synthesis and translation system is responsible for reviewing and synthesizing relevant research—both on innovations and on implementation—for the support and delivery systems. Schools must have the knowledge of evidence-based innovations and best practices for adapting such interventions, as well as the processes and factors that impact delivery of the innovation. As part of distilling and disseminating relevant literature, the synthesis and translation system is tasked with developing usable products (e.g., programs, resources, tools, and other materials) that guide the support and delivery systems in evidence-based practices. Several major frameworks offer insight into facilitators and barriers to implementation. CFIR identifies a set of contextual factors impacting implementation, including: individuals (e.g., leaders, deliverers, recipients), infrastructure and culture of the setting, and external pressures in impacting implementation effectiveness (Damschroder et al., 2022). Process models, such as GTO, guide delivery agents through needs assessment, goal development, program selection, capacity building, and evaluation (Wandersman et al., 2000). Understandings of implementation processes and determinants must be communicated across systems to promote successful implementation.

Broadly, a motivated synthesis and translation system is incentivized to create products that reflect the realities of support and delivery settings. The resources must be understandable, aligned with current priorities, include metrics of success, and offer guidance for effective rollout. The work of this system is largely in line with the efforts of implementation science to “promote the systematic uptake” of research into practice (Eccles & Mittman, 2006, p. 1). However, to demonstrate true readiness, the synthesis and translation system must move beyond efforts to publish research or develop manuals, to ensure that its output is aligned with the needs of the support and delivery systems.

It is essential for researchers, translators, and communicators to collaborate effectively within this system, and for outside partnerships to be established to ensure relevance of these products through the use of participatory research methods and being receptive to feedback. Integrated Knowledge Translation engages stakeholders in the research process in order to improve the likelihood of research’s inclusion in policy and practice. The Integrated Knowledge Translation approach includes an advisory research committee, a team with local partners to make research decisions, collaborative research and interpreting findings, and disseminating across reports, presentations, and journals (McIsaac et al., 2018). Efficacy relies upon synthesis and translation agents being able to define core components of SBH and operationalize implementation strategies, review and distill research, and translate scientific knowledge to lay language. A distillation and matching approach promotes synthesis of existing research to identify practice elements (i.e., discrete units of an intervention), compile a set of common elements (i.e., practice elements found across studies), and match sets of common elements to the intervention and recipient characteristics (Engell et al., 2023; Oddo et al., 2025). This system should have a champion to amplify the value of high quality synthesis and translation. The synthesis and translation system must value efforts to disseminate knowledge outside of traditional academic outlets. Successful efforts rely on sufficient funding, staffing, expertise, and time, as well as clear expectations and workflow for researchers and product developers. Readiness (motivation and capacity) is necessary for the ISF 2.0 systems to achieve their primary responsibilities. However, without readiness for interactions between systems, the efforts to effectively implement SBH programming will remain limited.

#### 4.2.4. System Interactions

While readiness within each system is crucial for dissemination and implementation to be successful, supporting each system individually is not enough. Rather than locating challenges solely within any individual system, the ISF 2.0 conceptualizes implementation as the product of three interdependent systems–synthesis and translation, support, and delivery–whose functioning and alignment jointly determine success. Thus, the ISF 2.0 emphasizes the importance of interactions across systems to accomplish outcomes. For instance, the synthesis and translation system is predominantly responsible for identifying and communicating evidence-based innovations to the support and delivery systems. However, the delivery system also plays a key role in sharing evaluation outcomes with the synthesis and translation system to further integrate into the evidence base. Each system, alone and working together, is instrumental in pushing the implementation and intervention fields forward.

Despite being conceptually interconnected, the synthesis and translation, support, and delivery systems often function in siloes. Yet, these systems do not exist in isolation, but rather interact in a broader societal and political context. If any system is excluded from external conversations (e.g., regarding funding), implementation efforts may be halted regardless of the systems’ internal interactions. Collective progress through implementation success relies on these systems acting as a cohesive unit, where knowledge, ideas, and progress are continuously flowing across all stakeholders (Aldridge et al., 2023). The systems-oriented structure aligns with the realities of SBH, where implementation success depends not only on the availability of evidence-based practices, but also on the presence of support infrastructures (e.g., training, coaching, data systems) and the capacity of schools and districts to deliver services within complex organizational and policy contexts (Forman et al., 2009).

Taken together, the ISF 2.0 offers an integrative lens for understanding why many evidence-based SBH practices fail in implementation and/or in achieving their intended outcomes, despite strong empirical support for the intervention. Table 1 summarizes key elements of successful SBH implementation and suggests corresponding analytic questions that can inform application of the ISF 2.0. The guiding questions were developed by the authors through the synthesis of SBH and implementation literature, described above. Recurring barriers, best-practice strategies, and gaps identified in the literature were summarized into key elements, organized by ISF 2.0 system, and distilled into preliminary analytic questions. The questions represent author-derived prompts grounded in the reviewed literature and mapped onto ISF 2.0 concepts, rather than additional components of the framework. We then applied the questions to three illustrative cases to examine strengths, gaps, and opportunities for improved functioning and alignment within and across systems. Although attempts were made to apply each question to each case example, we could not achieve perfect congruence because of variability in information available for each initiative. We present this illustration to demonstrate how the ISF 2.0 can show the strengths and gaps in current TTAC initiatives to advance SBH, as a necessary first step to enacting solutions.

## 5. Application of ISF 2.0: Three Illustrative Case Examples from SBH Training and Technical Assistance Centers

The ISF 2.0 can highlight challenges and promising practices across systems involved in SBH implementation efforts. To illustrate this application of the ISF 2.0, three cases of SBH TTACs were selected. Each case assesses an organization that focuses on SBH at state and/or national levels. We apply the ISF 2.0 to each case, describing each organization’s efforts within and across ISF 2.0 systems using the preliminary analytic questions (Table 1). Due to variability in available information on each initiative, questions were unable to be applied with perfect congruence across all case examples. We note which question(s) correspond to the content in our analysis below. Each case example details best practices from the organization, as well as gaps within and across ISF 2.0 systems. Additional information demonstrating the case example mapped across ISF 2.0 systems may be found in Supplementary File S1. Taken together, these examples illustrate how the ISF 2.0 may be used not only to map existing efforts but also to identify targeted strategies to enhance the coherence, scalability, and impact of SBH.

### 5.1. National Center for School Mental Health

Founded in 1995 at the University of Maryland, the National Center for School Mental Health (NCSMH) promotes national, state, and district efforts to implement school mental health programs through a combination of research, evaluation, training, technical assistance, policy, and advocacy initiatives (University of Maryland, 2026).

#### 5.1.1. Synthesis and Translation

The NCSMH developed the School Health Assessment and Performance Evaluation (SHAPE) System, offering free, publicly accessible resources to enhance school mental health quality (https://theshapesystem.com/). The SHAPE system houses and organizes hundreds of resources, including assessments and guidance materials for implementing SBH practices (Table 1, question 3a). For instance, the School Mental Health Quality Guides distill research into best practices for supporting implementation of teaming, needs assessment and resource mapping, screening, mental health promotion, and early intervention and treatment (Table 1, question 3b). They also support navigating funding and sustainability and documenting the impact of comprehensive school mental health systems. To expand dissemination efforts, the NCSMH previously partnered with the SAMHSA-funded Mental Health Technology Transfer Center (MHTTC) to develop and disseminate implementation guidance modules aligning with the quality guides, which continue to be publicly accessible via Stanford University (Table 1, question 4a; Stanford Medicine, n.d.).

#### 5.1.2. Implementation Support

Beyond synthesis and translation efforts, the NCSMH demonstrates the support system in action through numerous training and technical assistance initiatives. These efforts include a national learning collaborative (Bohnenkamp et al., 2024) and providing TTA around SBH topics in partnership with state agencies (e.g., Maryland State Department of Education), national professional associations (e.g., National Association of School Nurses), school districts, and regional and local community organizations (Table 1, question 2b). Through the NCSMH Learning Collaborative, national leaders interacted with state-level education and behavioral health representatives and individuals in the delivery system (e.g., district school mental health teams) to support the implementation, scale-up, and improvement of comprehensive SBH systems (Table 1, questions 1b, 2a; Bohnenkamp et al., 2024). Support system activities included orientation webinars, state leadership training and kickoff meetings, coaching of state leads, virtual learning sessions centered around quality improvement (QI) and SBH best practices, monthly action period calls focused on application of QI cycles, monthly office hours for state leads, ongoing email and web support, and access to tools and software to facilitate local change efforts (Table 1, question 2b). A recent evaluation of this learning collaborative provided support for both the feasibility and impact of this approach to implementation support (Table 1, question 2e; Bohnenkamp et al., 2024).

#### 5.1.3. Cross-System Interaction

The NCSMH has multiple channels of direct interaction with the delivery system (Table 1, question 4a). In addition to their large national conference that brings together researchers, policymakers, and practitioners, the NCSMH directly supports SBH programs in local schools. This takes place through funding direct service interventions and providing supervision to satisfy practice requirements. These efforts promote high-quality service delivery and facilitate workforce development. The NCSMH also interacts with a large community of practitioners through their listserv to share policy updates, open school-based positions, TTA, resources, grants/research opportunities.

The NCSMH further exemplifies engagement with the larger societal systems identified through the ISF 2.0, namely, research and policy (Table 1, question 4d). The NCSMH has a team of in-house and collaborating research faculty and staff leading more than a dozen active or recently completed grant-funded projects. The projects include topics such as expanding access to SBH services through partnerships and technological advancements, developing measures, supporting and evaluating state initiatives, enhancing mental health literacy delivery in local schools, and pioneering culturally responsive approaches to addressing workforce retention challenges (University of Maryland, 2026). NCSMH interweaves their synthesis and translation, support, and research efforts by providing TTA and evaluation support in many of these projects (Table 1, questions 4a-c). Further, the NCSMH actively interfaces with policy in multiple ways (Table 1, question 4d). As a consumer of policy, the NCSMH reviews, distills, and disseminates information on policies relevant to SBH through their interactive policy map in the SHAPE system. The Center also seeks to drive policy through collaborations with national organizations (e.g., Hopeful Futures Campaign, Council of Chief State School Officers, and Association of State and Territorial Health Officials) and sharing advocacy resources via their website (University of Maryland, 2026).

#### 5.1.4. Opportunities for Enhanced Alignment

The NCSMH demonstrates how a large, national center rooted in a university can directly engage in and/or facilitate efforts across all ISF 2.0 systems. While specific initiatives aim to effect change in multiple readiness components, the NCSMH’s efforts skew toward building innovation-specific capacity (Table 1, question 3b). The SHAPE system does include some resources, such as the Organizational Well-Being Inventory for Schools (OWBI-S; Hoover et al., 2023), that address general capacity and motivation. However, most of the quality guides and other resources focus on assessing and delivering specific school mental health practices (Table 1, question 3b). Further, it is unclear how exactly the synthesis and translation and support systems interact outside of specific initiatives such as the learning collaborative reviewed above, as well as pathways for systems to communicate upstream (e.g., from the delivery system to the support and synthesis and translation systems; Table 1, questions 4a–c). This suggests opportunities to bolster and/or make more explicit these interactions to strengthen alignment and improve reach and impact.

### 5.2. Center on Positive Behavioral Interventions and Supports

The Center on PBIS is a national technical assistance center funded by the U.S. Department of Education focused on promoting implementation of PBIS at the regional, state, district, and school level (Center on PBIS, 2026).

#### 5.2.1. Synthesis and Translation

The center has developed and published numerous implementation resources for various audiences. These include fidelity inventories and assessments for the evaluation of readiness and implementation outcomes; blueprints for implementing the comprehensive PBIS framework (at district and school levels); practice briefs and guides for specific PBIS components; readymade tools for educators, practitioners, and leaders (e.g., syllabi, lesson plans, and templates for meetings and action plans); and examples from the field (Table 1, question 3a). While most resources are intended for individuals in the delivery system, the center has also produced resources for the support system (e.g., coaching materials) and policymakers (e.g., policy briefs). Dozens of presentation materials and recorded webinars and trainings are available to foster asynchronous learning on a variety of topics (Table 1, question 3a). Much of the material synthesized and translated by the center targets innovation-specific capacity, unpacking specific PBIS practices, such as interventions, screeners, and progress monitoring tools. However, the center’s commitment to implementation has also yielded resources for building the general capacities that underlie PBIS implementation, such as leadership, teaming, and the implementation process (Table 1, question 3b).

#### 5.2.2. Implementation Support

Somewhat less visible is the Center on PBIS’s role in developing a support system to facilitate PBIS implementation. The center advocates a cascade model interconnecting key partners at different levels. In addition to a team of national co-directors, advisors, and partners, the center interacts with multiple regional networks (e.g., Midwest PBIS, Northeast PBIS) and state coordinators to disseminate information and resources from the national level to individual school districts (Table 1, question 2b). The center has long promoted coaching at the individual through systems levels to support PBIS implementation. This involves regional and/or state PBIS leadership teams providing external support to district PBIS teams, who are responsible for action planning, funding, visibility, political support, policy, and building personnel readiness (Sugai & LaMaster, 2015). The district PBIS team provides support to school PBIS teams around action and data planning, leadership, training, coaching, and evaluation; then, the school PBIS team delivers internal coaching to school staff to guide implementation of action plans and specific PBIS practices (Sugai & LaMaster, 2015). In proposing this cascade of support, the Center on PBIS outlines a multi-level support infrastructure with defined roles and functions. They further support the adoption of this model and the delivery of coaching through a practice brief (Duda & Barrett, 2013) and coaching tools like the Coaching Inventory Discussion Tool (Duda & Barrett, 2014) and the Coaches Self-Assessment (Lewis-Palmer et al., 2004), all available on their website (Table 1, question 2d).

The Midwest PBIS Network, a regional hub affiliated with the Center on PBIS, has emerged as a leader in supporting implementation of PBIS. Operating as a non-profit, Midwest PBIS has extended the Center on PBIS’s synthesis and translation efforts by creating a publicly available repository of tools and resources that coaches and school and district personnel can use to enhance their implementation of comprehensive SBH (Midwest PBIS, n.d.). Through their website, coaches and individuals in the delivery system can access additional informational and implementation resources (e.g., guides, templates, samples) and numerous recorded webinars and trainings on targeted topics, or sign up for virtual or in-person training packages (Table 1, question 4c). Midwest PBIS employs coaches that provide implementation support directly to districts and schools (with multiple coaching packages available at varying price points) and promotes the growth of the coaching workforce and the delivery of high-quality coaching through coaching guidance material and networking opportunities (Table 1, questions 2a–b).

#### 5.2.3. Cross-System Interaction

In addition to the many resources developed by the center (all of which are freely available through their website), the Center on PBIS organizes annual conferences to bring together researchers, leaders, educators, and practitioners to advance PBIS (Table 1, questions 4a–b). Their poster session specifically centers individuals in the delivery system demonstrating strong examples of PBIS to facilitate the transfer of information horizontally within the delivery system (between schools and districts) and vertically to individuals engaged in support, synthesis and translation, and research (Table 1, questions 1a–b, 4a–c). Thus, the center facilitates bidirectional communication across systems and intentionally opens channels for community-based evidence to travel upstream (Table 1, questions 4a–c).

The Center on PBIS illustrates engagement with broader societal structures through its longstanding federal funding and alignment with national education priorities. As a federally funded technical assistance center, its activities are shaped by and responsive to evolving policy emphases (e.g., school safety, climate, and student well-being), enabling the center to remain relevant and influential at scale (Table 1, question 4d). In turn, the Center on PBIS contributes to shaping practice and policy by disseminating guidance, tools, and frameworks that are frequently adopted or adapted by state education agencies and districts, thereby influencing downstream policy (Table 1, question 4d).

#### 5.2.4. Opportunities for Enhanced Alignment

The Center on PBIS robustly spans synthesis and translation, implementation support, and engagement with the delivery system; however, the ISF 2.0 highlights several opportunities to strengthen cross-system alignment. First, although the center has developed an extensive body of evidence-based synthesis and translation products (e.g., tools and training for specific PBIS components and quality improvement), it is less clear on how support systems should use these resources (Table 1, question 4b). Further, these resources and guidance are likely variable across contexts, given the field’s reliance on regional and state intermediaries. In other words, although the center has invested in building high-level infrastructure to promote technical assistance (via coaching), there is an opportunity to ramp up TA efforts beyond the few coaching briefs and tools currently available. This might include expanding coaching-specific resources (e.g., practice profiles, session protocols, exemplar case examples), articulating a tiered coaching model aligned with PBIS implementation stages, developing tools to guide data-informed coaching interactions, and building more formalized training and credentialing opportunities for coaches (Table 1, questions 2b, 2d, 4b). In addition, PBIS resources remain largely oriented toward building innovation-specific capacity (e.g., fidelity to PBIS practices and data systems), with comparatively less emphasis on developing general capacity (e.g., leadership, organizational functioning) and motivation (Table 1, question 3b). Together, these patterns suggest a need for clearer linkage between synthesis and translation products and support system activities (e.g., practice profiles, crosswalks linking tools to coaching actions).

The ISF 2.0 further draws attention to opportunities to enhance bidirectional communication and integration across systems. While the center creates important spaces for exchange (e.g., national forums), mechanisms for systematically capturing and incorporating ongoing feedback from the delivery system into synthesis, translation, and support efforts are unclear (Table 1, questions 4a, 4c). Strengthening the collection and use of practice-based evidence could support more continuous adaptation of resources and supports to local needs (L. W. Green, 2008). Finally, the cascade model of support, while enabling broad dissemination, may benefit from greater investment in internal capacity building (e.g., development of local coaching infrastructure) to promote sustainability and reduce reliance on external expertise (Table 1, question 2c–d). Enhancing local technical assistance delivery, a recognized implementation strategy (Cook et al., 2019), may help close the gap between PBIS reach (over 27,000 schools; Center on PBIS, 2026) and quality (McIntosh et al., 2017).

### 5.3. South Carolina School Behavioral Health Academy

The youngest of these centers, the South Carolina School Behavioral Health Academy (SBHA; Coates et al., 2024) was established in 2022 in partnership between leaders at the University of South Carolina and the SC Department of Health and Human Services (DHHS). The SBHA was created to expand SBH workforce capacity through a combination of high-quality training available to all and tailored coaching to school districts. By increasing the accessibility of SBH best practices (through the free, online training system and associated app) and developing a formal support system, the SBHA aims to enhance the capacity of the entire educational system (educators, specialists, non-specialists, leaders) to prevent and respond to student behavioral health needs. The following information regarding SBHA was obtained through published articles, the SBHA website, and direct observations and experiences from the author associated with this initiative.

#### 5.3.1. Synthesis and Translation

The SBHA synthesizes, translates, and disseminates best practices in SBH through its learning management system (LMS; Coates et al., 2024). The LMS provides free, virtual access to over 43 h of asynchronous, interactive training on SBH best practices (Table 1, question 3a). The LMS courses largely focus on developing knowledge and skills to deliver specific SBH practices. For instance, content includes the elements of evidence-based practices, tools for screening and monitoring progress, how to match interventions to student needs, and how to analyze and interpret data to make sound decisions based on intervention response (South Carolina School Behavioral Health Academy, 2024). In ISF 2.0 terms, most courses aim to enhance the innovation-specific capacity, particularly knowledge and skills, of educators and practitioners in the delivery system (Table 1, question 3b). One course (“Core MTSS Practices”) centers the general capacities that underlie effective SBH implementation: leadership, teaming, needs assessment and resource mapping. Based on knowledge from the second author, the course catalog is in continual development, with a multi-disciplinary team of content experts from research and practice backgrounds drafting new courses and revising existing courses annually (Table 1, question 3c).

The LMS not only synthesizes research into training content and resources on SBH but also emphasizes translating information into usable materials for a wide audience in the delivery system (Table 1, question 3a). Informed by best practices in adult learning and professional development, the LMS includes numerous interactive features, experiential scenarios, real examples from the field, readymade printable resources that distill training content, and frequent opportunities to review and apply knowledge. Learners can progress at their own pace and choose whether to access linked resources for deeper learning on relevant topics.

#### 5.3.2. Implementation Support

The second arm of the SBHA is the coaching program, which takes a systems coaching approach (Pierce, 2025), primarily focused on supporting district-level change in SBH through access to free coaching (Table 1, question 2b; Perkins et al., 2026). As part of the participation agreement, districts identify pilot schools that also receive coaching support to guide them through the SBH implementation process and provide a model for the district to replicate (Table 1, question 2a). Districts receive ongoing, tailored support from an assigned coach (Table 1, question 2c). SBHA coaches support team startup and teaming processes; educate and build investment from district leaders; train staff and connect teams to SBH resources; and facilitate system-wide needs assessment, resource mapping, goal setting, action planning, and the collection and use of data for continuous improvement (Table 1, question 2b; Perkins et al., 2026). Through coaching sessions and a coaching community of practice, coaches focus on building the general capacity of district and school teams to sustain the framework independently (Table 1, question 2d; Perkins et al., 2026). This includes intentionally cultivating understanding of implementation and improvement processes, and implementation support specifically.

#### 5.3.3. Cross-System Interaction

The SBHA demonstrates the bidirectional transfer of information between ISF 2.0 systems. Information flows from the synthesis and translation system to the delivery system via the LMS, and from the support system to the delivery system via the coaching program (Table 1, questions 4a, 4c). The organization of the SBHA, being housed within a university center, enables frequent and real-time interaction between synthesis and translation and support systems, with overlapping membership and monthly whole-team meetings (based on knowledge from the second author; Table 1, question 4b). Additionally, the SBHA establishes pathways for LMS participants in the delivery system to communicate directly with the SBHA team through feedback forms and buttons for submitting inquiries (Table 1, question 4a). Delivery system providers can interact with the SBHA team in real-time by attending the SBHA’s free quarterly webinars (Table 1, question 4a). Informal feedback is also continually sought from recipients of coaching (namely, district and school leaders; Table 1, question 4b; Perkins et al., 2026). The information and feedback received by LMS participants and coaching recipients informs revisions to existing LMS courses, resource creation, and the development of supplemental courses (Table 1, questions 3c–d).

The SBHA further illustrates how each ISF 2.0 system interacts with broader societal structures. Funded by a state agency (the South Carolina Department of Health and Human Services (SCDHHS), the project was designed to support and expand the workforce (Perkins et al., 2026). This shapes the SBHA’s priorities and situates its efforts firmly within the ISF 2.0 systems interconnecting research and practice (as opposed to focusing on either end of the continuum; Table 1, question 4d). Yet, because the SBHA was developed in partnership with researchers at the University of South Carolina, it has access to research expertise and resources that similar initiatives founded and funded through different mechanisms might lack. The longstanding cross-sector professional relationships that precipitated the SBHA also informed its efforts, such as allowing for compromise between focusing on expanding the clinical workforce and emphasizing systems-oriented work and implementation support. The nature of this partnership created the backdrop for the SBHA to be responsive to multiple pressing needs (as opposed to constrained by a funder with a more singular focus) and facilitated connection to a wider audience than would have been otherwise reached.

#### 5.3.4. Opportunities for Enhanced Alignment

In sum, the SBHA illustrates efforts within the ISF 2.0 synthesis and translation and support systems, as well as the interaction between these systems and the delivery system. The ISF 2.0 can also be applied to analyze gaps within and between these systems and identify potential solutions. For instance, while there are established channels for communicating between the synthesis and translation system and the delivery system (through optional feedback forms embedded in the LMS) and between the support system and delivery system (via coaching sessions), this interaction could be more systematic and/or robust (Table 1, questions 4a, 4c). SBHA could benefit from a centralized system for capturing and integrating feedback received through coaching and the LMS; expanding cross-site collaborative learning structures (e.g., communities of practice or learning collaboratives) to surface common challenges and solutions; and/or creating explicit mechanisms for making feedback-informed adaptations and communicating changes back to stakeholders (Table 1, questions 1d, 2e, 3c–d).

ISF 2.0 also suggests that efforts between systems may not always be congruent. For instance, the LMS (synthesis and translation system) is more innovation-focused, whereas coaching efforts (support system) balance innovation-specific capacity, general capacity, and motivation (Table 1, question 3b). While blueprints exist for implementing SBH, similar guidance is not yet available for building general capacity and motivation in this field, leaving coaches and delivery system providers (school leaders, educators, and practitioners) without a map for developing key elements of readiness. Moreover, the infrastructure connecting support and delivery systems could be further developed. Although the SBHA coaching program demonstrates promising results (Perkins et al., 2026), the limited size of the coaching team may constrain capacity to significantly scale up coaching services. Therefore, SBHA may benefit from bolstering internal support systems in which district- and/or school-level coaches, coordinators, or other leaders provide ongoing support (Table 1, question 2d). Of course, this requires that internal support providers have the necessary knowledge and skills. While the SBHA facilitates internal coach development through modeling and annual coaching institutes, these initiatives are only available to participating districts. Developing a LMS module specifically geared toward coaching and creating resources and tools that define the coaching model and specify key coaching practices may facilitate broader scale-up of coaching (Table 1, question 4b).

Taken together, these case examples illustrate how the ISF 2.0 can be used not only to map existing strengths, but also to identify targeted strategies to enhance coherence, scalability, and impact of SBH.

## 6. Discussion

The systemic uptake of research into practice remains a persistent challenge, despite decades of implementation research aimed at improving this process (Rapport et al., 2018; Westerlund et al., 2019). Although implementation science has advanced our understanding of barriers and facilitators of implementation, SBH efforts continue to face difficulty in integrating effective and sustainable programming into school environments. Our critical narrative review suggests that successful implementation depends not only on the availability of effective innovations but also on how well the systems responsible for synthesizing and translating evidence, providing implementation support, and delivering services work together. When these systems are aligned, they may be better positioned to identify evidence-based programs that fit the local context, provide training and ongoing support to delivery agents, and evaluate outcomes.

In the present article, we used the ISF 2.0 as an analytical lens to identify several factors that may contribute to these persistent challenges across three illustrative examples. First, there appears to be a tendency to emphasize innovation-specific capacity while giving less attention to other components of readiness, including motivation and general capacity. This imbalance may contribute to difficulties sustaining evidence-based programming over time. Second, consistent with prior work (Wandersman et al., 2024), our case analyses suggest that the support system is often underdeveloped and fragmented. In the absence of a shared model for implementation support, approaches may vary considerably across states, initiatives, and organizations. Limited formal training pathways for support providers may constrain the field by emphasizing the training of delivery agents while giving less attention to building the capacities of those who provide technical assistance and other forms of implementation support. Lastly, a diffusion of responsibility can make it unclear who owns cross-system collaboration and key aspects of the implementation processes. The resulting lack of accountability may contribute to incongruence across key functions, such as synthesis, translation, training, support, evaluation, and coordination.

Taken together, these findings support the proposition that the alignment across systems is central to effective implementation. Accordingly, the presented conceptual application of the ISF 2.0 illustrates its potential as a practical framework for identifying cross-system breakdowns and guiding the coordinated readiness-building needed to improve SBH implementation and, ultimately, individual and community outcomes.

### 6.1. Limitations

Several limitations should be considered when interpreting these findings and identifying directions for future research. First, the review was narrative and not systematic and should be viewed as an illustrative, rather than an exhaustive overview of the SBH and implementation literature. Second, the three case examples were selected based on national recognition, accessibility of information, and familiarity with the initiatives. One author has a direct connection to the South Carolina School Behavioral Health Academy (SBHA), which may have introduced selection and interpretation bias. Although this insider knowledge facilitated a more in-depth analysis of this case, it may also have influenced which evidence was emphasized and how it was interpreted. Accordingly, the cases should not necessarily be viewed as representative of all SBH implementation efforts; rather, they offer several prominent examples that help illustrate the potential of the ISF 2.0 as a diagnostic lens. Furthermore, this review conceptually considers the utility of the ISF 2.0 for evaluating and promoting SBH implementation. However, its use in this way was not empirically tested to confirm that the ISF 2.0 improves implementation outcomes, system alignments, or sustainability.

Finally, the ISF 2.0 emphasizes bidirectional relationships among systems, making it important to acknowledge our own positionality and the limited representation from stakeholders outside of academia. The authors are predominantly researchers housed within academic institutions, although several have clinical training and have experience working in support and delivery systems. As a result, the article primarily reflects perspectives from researchers and the synthesis and translation system. These perspectives provide important expertise, but they do not capture the experiences and priorities of school staff, students, families, community members and other stakeholders whose engagement is essential to the real-world use of the ISF 2.0. Future work should strengthen representation of these partners and ensure that multiple perspectives inform implementation research and practice.

### 6.2. Future Research Directions

A key direction for future research is to further develop and test the ISF 2.0 as a diagnostic tool for strengthening SBH implementation. This work should move beyond demonstrating the usefulness of ISF 2.0-guided questions to examining whether systematic use of the framework improves system alignment, implementation outcomes, and sustainability. Research should also establish a more structured diagnostic process or tool that can be used consistently across settings while remaining adaptable to local contexts. Importantly, this tool or process would benefit from guiding the evaluation and promotion of readiness components within and between systems. Towards this future direction, we aim to garner expertise from across the systems to develop a tool that is usable and meets the needs of the stakeholders in the ISF. In this way, the process of tool development may itself demonstrate an approach of how to work across systems in collaborative partnerships.

The ISF 2.0 emphasizes that implementation is shaped by interactions among systems and is most likely to succeed when systems work together towards shared goals. This perspective suggests several additional priorities. Although implementation science increased understanding of barriers and facilitators to implementation (Eccles & Mittman, 2006), the field may still place disproportionate emphasis on innovation and innovation-specific capacity. The emphasis may overshadow the general capacities needed to support dissemination, translation, implementation, evaluation, and quality improvement. As a result, schools and other delivery systems may lack the infrastructure and skills needed to evaluate outcomes consistent with best practices (Bartlett et al., 2017). Future efforts should place greater emphasis on identifying and synthesizing common elements across SBH interventions (Oddo et al., 2025) and implementation frameworks (Engell et al., 2023). Greater attention to shared implementation ingredients could facilitate a common language across the synthesis and translation, support, and delivery system and make it easier to identify the capacities required for successful implementation. This knowledge should be paired with accessible and contextually appropriate evaluation tools that enable schools and other delivery settings to generate practice-based evidence and contribute to the broader evidence base.

Another priority is strengthening the support system, which serves as a bridge between synthesis and translation and delivery systems. Research is needed to strengthen the support system’s capacity to assess and communicate its own needs to the synthesis and translation system, evaluate the quality and effectiveness of training and technical assistance efforts, and work collaboratively with the delivery systems to assess program implementation and outcomes. Developing and testing models for capacity-building within the support system may be particularly important for creating the sustained infrastructure needed to move evidence into practice.

### 6.3. Driving Solutions: A Call to Action

As the ISF 2.0 demonstrates, solutions to dissemination and implementation failures do not exist within a single system. Rather, systems must be equipped to fulfill their own roles and responsibilities, as well as to collaborate across their domains. In this article, we aimed to clarify the roles and responsibilities (e.g., illustrate readiness) within each system, while emphasizing the need for readiness to align between systems. We call on individuals housed in or functioning on behalf of each system to take on this work. Best said nearly two decades ago: “If we keep on doing what we have been doing, we are going to keep on getting what we have been getting” (Wandersman et al., 2008, p. 171). Change can only occur with systems that are willing and able to take action against the status quo. Thus, we offer several avenues for such action.

#### 6.3.1. Delivery System

Delivery systems would benefit from demanding increased accountability from the support system, including ongoing requests for training and technical assistance, evaluation of training, etc. Within the delivery system, readiness involves the adequate evaluation and quality improvement of services. Thus, delivery agents must be prepared to collaborate with support and synthesis and translation systems to ensure that findings from their efforts are effectively incorporated into the evidence base. In so doing, the delivery system is called to engage as active partners in–rather than passive recipients of–knowledge creation, evaluation, and dissemination.

#### 6.3.2. Support System

The support system must call on the synthesis and translation system to develop usable tools to be better equipped to handle the demand from the delivery system. As previously described, the support system needs to engage in self-evaluation to identify needs, gaps, and areas for quality improvement. Such efforts may be best guided by improved understanding of dissemination and implementation principles to ensure that the support system espouses best-practice recommendations in their own initiatives and in the support provided to delivery systems.

#### 6.3.3. Synthesis and Translation System

Perhaps most importantly to researchers, the synthesis and translation system must shift to a more collaborative approach, such as community-based participatory research. Researchers must intentionally cultivate their ability to effectively communicate science, be it through graduate training, direct experience in delivery systems, or other pathways. Thus, driving solutions calls for practices within and between systems to be examined and for this awareness to facilitate a movement of change. While the ISF 2.0 emphasizes the bidirectionality of knowledge across systems, we posit that the driving force of these changes relies on researchers and others within the synthesis and translation system. However, in order to guide these changes, the synthesis and translation system would benefit from clarified expectations to bolster the work of the support system (e.g., translate implementation science into usable guidance) and partner with the delivery system to ensure resources are usable.

#### 6.3.4. Cross-System Collaboration

We further call for enhancing the capacity for collaboration across all systems. There is a need for field-wide training in dissemination and implementation, collaboration and team science (e.g., community-based participatory research), and evaluation and QI. Each system must have the requisite knowledge to engage in these efforts. Cross-system collaboration would benefit from improved incentives from funders, academia, and broader societal structures to enhance motivation. Such external incentives and oversight have the potential to improve outcomes and further drive internalized motivation and sustained collaboration. The ISF 2.0 offers a conceptual framework to drive these efforts forward, as well as a diagnostic approach that can inform ongoing evaluation and quality improvement.

## 7. Conclusions

The present article reviewed current best practices and limitations in SBH implementation. Using a systems-level implementation framework as an analytical lens, we identified strengths, gaps, and opportunities across systems responsible for SBH research, implementation support, and delivery. Based on a guiding set of questions, informed by the ISF 2.0, we demonstrated the potential of an expanded systems approach for diagnosing barriers and guiding solutions across prominent examples of SBH implementation efforts. Our review highlighted ideas on *how* to do something different; however, change cannot occur without action from each system. We call on SBH stakeholders across systems to enhance readiness and cross-system collaboration. The author team aims to foster such efforts through ongoing development of a diagnostic tool or process that may afford funders, researchers, and support staff the opportunity to look at the presence and absence of key readiness ingredients within and between systems. Through collaborative partnerships, we have the potential to translate implementation science into usable guidance, develop evaluation tools and methods that are feasible for the community, and spearhead the intentional alignment of efforts across systems to maximize the effectiveness of school behavioral health efforts.

## Figures and Tables

**Table 1:** Key Principles and Questions for Examining SBH Implementation Through the ISF 2.0.

System	Key Elements/Principles	Guiding Questions for Case Analysis
Delivery	Partnerships and collaboration Readiness (motivation and capacity) Continuous evaluation	(1a) How are schools engaged? (1b) How are partnerships built? (1c) How is readiness developed? (1d) How are implementation and outcomes evaluated and used for improvement?
Support	Partnerships Continuum of training and technical assistance Ongoing, tailored support Capacity building Evaluation	(2a) How is support accessed and delivered? (2b) What options for support are available? (2c) How is ongoing support sustained? (2d) How does support build delivery-system capacity? (2e) How is support evaluated?
Synthesis and Translation	Usable products Contextual factors Implementation and improvement processes Interprofessional collaboration and stakeholder engagement	(3a) How is evidence translated into usable products? (3b) Do products address all aspects of readiness in delivery and support systems? (3c) How is feedback used to inform future products? (3d) How are delivery/support system providers involved in product development and improvement?
Cross-system	Communication Feedback Coordination Alignment	(4a) How does the synthesis and translation system communicate with the delivery system? (4b) How does the synthesis and translation system communicate with the support system? (4c) How does the support system communicate with the delivery system? (4d) How do systems interact with research, societal structures, and the public?

Note. Guiding questions were developed by the authors through a synthesis of the SBH and implementation literature and organized according to ISF 2.0 systems. Questions represent preliminary analytic prompts rather than additional ISF 2.0 constructs.

## Data Availability

No new data were created or analyzed in this study. Data sharing is not applicable to this article.

## Supplementary Materials

The following supporting information can be downloaded at: https://www.mdpi.com/article/10.3390/bs16091663/s1.

## Abbreviations

The following abbreviations are used in this manuscript:

SBH	School Behavioral Health
ISF	Interactive Systems Framework for Dissemination and Implementation
EBP	Evidence-Based Practice
TTA	Training and technical assistance
CBPR	Community-based participatory research
CFIR	Consolidated Framework for Implementation Research
GTO	Getting To Outcomes
IKT	Integrated knowledge translation
QI	Quality Improvement
NCSMH	National Center for School Mental Health
PBIS	Positive Behavioral Interventions and Supports
SBHA	School Behavioral Health Academy
LMS	Learning management system

## References

[B1-behavsci-16-01663] Albers B., Mildon R., Lyon A. R., Shlonsky A. (2017). Implementation frameworks in child, youth and family services—Results from a scoping review. Children and Youth Services Review.

[B2-behavsci-16-01663] Aldridge W. A., Roppolo R. H., Brown J., Bumbarger B. K., Boothroyd R. I. (2023). Mechanisms of change in external implementation support: A conceptual model and case examples to guide research and practice. Implementation Research and Practice.

[B3-behavsci-16-01663] Baffsky R., Ivers R., Cullen P., Wang J., McGillivray L., Torok M. (2023). Strategies for enhancing the implementation of universal mental health prevention programs in schools: A systematic review. Prevention Science.

[B4-behavsci-16-01663] Balis L. E., Palmer S., Isack M., Yaroch A. L. (2025). Barriers and facilitators to program evaluation and dissemination: A qualitative study to inform implementation strategies. Health Promotion Practice.

[B5-behavsci-16-01663] Barrett S., Eber L., Weist M. D. (2013). Advancing education effectiveness: An interconnected systems framework for positive behavioral interventions and supports (PBIS) and school mental health.

[B6-behavsci-16-01663] Bartlett R., Wright T., Olarinde T., Holmes T., Beamon E. R., Wallace D. (2017). Schools as sites for recruiting participants and implementing research. Journal of Community Health Nursing.

[B7-behavsci-16-01663] Beidas R. S., Boyd M., Casline E., Scott K., Patel-Syed Z., Mills C., Mustanski B., Schriger S., Williams F. S., Waller C., Helseth S. A., Becker S. J. (2025). Harnessing implementation science in clinical psychology: Past, present, and future. Annual Review of Clinical Psychology.

[B8-behavsci-16-01663] Bennett K., Manassis K., Duda S., Bagnell A., Bernstein G. A., Garland E. J., Miller L. D., Newton A., Thabane L., Wilansky P. (2015). Preventing child and adolescent anxiety disorders: Overview of systematic review. Depression and Anxiety.

[B9-behavsci-16-01663] Bohnenkamp J., Robinson P., Connors E., Carter T., Orenstein S., Reaves S., Hoover S., Lever N. (2024). Improving school mental health via national learning collaboratives with state and local teams: Components, feasibility, and initial impacts. Evaluation & the Health Professions.

[B10-behavsci-16-01663] Boothroyd R. I., Flint A. Y., Lapiz A. M., Lyons S., Jarboe K. L., Aldridge W. A. (2017). Active involved community partnerships: Co-creating implementation infrastructure for getting to and sustaining social impact. Translational Behavioral Medicine.

[B11-behavsci-16-01663] Brann K. L., Naser S. C., Splett J. W., DiOrio C. A. (2021). A mixed-method analysis of the implementation process of universal screening in a tiered health system. Psychology in the Schools.

[B12-behavsci-16-01663] Bruhn A. L., Lane K. L., Hirsch S. E. (2014). A review of tier 2 interventions conducted within multitiered models of behavioral prevention. Journal of Emotional and Behavioral Disorders.

[B13-behavsci-16-01663] Bumbarger B. K., Moore J. E., Crane M. E. (2024). Evidence-based implementation support: Considering motivation and capacity within the ecosystem of training and technical assistance. Evaluation and the Health Professions.

[B14-behavsci-16-01663] Center on PBIS (2026). Back to school 2026!.

[B15-behavsci-16-01663] Cho Blair K. S., Park E. Y., Kim W. H. (2021). A meta-analysis of Tier 2 interventions implemented within school-wide positive behavioral interventions and supports. Psychology in the Schools.

[B16-behavsci-16-01663] Coates A. G., Knott H., Collins D., Parrish L., McQuillin S. D., Weist M. D. (2024). The South Carolina school behavioral health academy. Communique.

[B17-behavsci-16-01663] Collier-Meek M. A., Fallon L. M., DeFouw E. R. (2017). Toward feasible implementation support: E-mailed prompts to promote teachers’ treatment integrity. School Psychology Review.

[B18-behavsci-16-01663] Collier-Meek M. A., Sanetti L. M. H., Boyle A. M. (2016). Providing feasible implementation support: Direct training and implementation planning in consultation. School Psychology Forum: Research in Practice.

[B19-behavsci-16-01663] Collins S. E., Clifasefi S. L., Stanton J., Straits K. J., Gil-Kashiwabara E., Rodriguez Espinosa P., Nicasio A. V., Andrasik M. P., Hawes S. M., Miller K. A., Nelson L. A., Orfaly V. E., Duran B. M., Wallerstein N. (2018). Community-based participatory research (CBPR): Towards equitable involvement of community in psychology research. American Psychologist.

[B20-behavsci-16-01663] Connors E. H., Smith-Millman M., Bohnenkamp J. H., Carter T., Lever N., Hoover S. A. (2020). Can we move the needle on school mental health quality through systematic quality improvement collaboratives?. School Mental Health.

[B21-behavsci-16-01663] Cook C. R., Lyon A. R., Locke J., Waltz T., Powell B. J. (2019). Adapting a compilation of implementation strategies to advance school-based implementation research and practice. Prevention Science.

[B22-behavsci-16-01663] Damschroder L. J., Reardon C. M., Opra Widerquist M. A., Lowery J. (2022). Conceptualizing outcomes for use with the consolidated framework for implementation research (CFIR): The CFIR outcomes addendum. Implementation Science.

[B23-behavsci-16-01663] Duda M. A., Barrett S. (2013). Coaching for competence and impact. Brief 1: Defining coaching.

[B24-behavsci-16-01663] Duda M. A., Barrett S. (2014). Coaching for competence and impact: Coaching inventory discussion tool.

[B25-behavsci-16-01663] Durlak J. A., DuPre E. P. (2008). Implementation matters: A review of research on the influence of implementation on program outcomes and the factors affecting implementation. American Journal of Community Psychology.

[B26-behavsci-16-01663] Durlak J. A., Mahoney J. L., Boyle A. E. (2022). What we know, and what we need to find out about universal, school-based social and emotional learning programs for children and adolescents: A review of meta-analyses and directions for future research. Psychological Bulletin.

[B27-behavsci-16-01663] Durlak J. A., Weissberg R. P., Dymnicki A. B., Taylor R. D., Schellinger K. B. (2011). The impact of enhancing students’ social and emotional learning: A meta-analysis of school-based universal interventions. Child Development.

[B28-behavsci-16-01663] Eccles M. P., Mittman B. S. (2006). Welcome to implementation science. Implementation Science.

[B29-behavsci-16-01663] Engell T., Stadnick N. A., Aarons G. A., Barnett M. L. (2023). Common elements approaches to implementation research and practice: Methods and integration with intervention science. Global Implementation Research and Applications.

[B30-behavsci-16-01663] Ennis R. P., Royer D. J., Lane K. L., Dunlap K. D. (2020). The impact of coaching on teacher-delivered behavior-specific praise in Pre-K-12 settings: A systematic review. Behavioral Disorders.

[B31-behavsci-16-01663] Evans S. W., Randy Koch J., Brady C., Meszaros P., Sadler J. (2013). Community and school mental health professionals’ knowledge and use of evidence based substance use prevention programs. Administration and Policy in Mental Health and Mental Health Services Research.

[B32-behavsci-16-01663] Evans S. W., Simonsen B., Dolan G., Barrett S., Eber L., Weist M. (2017). School level practices. Advancing education effectiveness: Interconnecting school mental health and school-wide positive behavior support.

[B33-behavsci-16-01663] Fetterman D., Rodríguez-Campos L., Wandersman A., O’Sullivan R. G. (2014). Collaborative, participatory, and empowerment evaluation: Building a strong conceptual foundation for stakeholder involvement approaches to evaluation (A response to Cousins, Whitmore, and Shulha, 2013). American Journal of Evaluation.

[B34-behavsci-16-01663] Fetterman D. M., Wandersman A. (2005). Empowerment evaluation principles in practice.

[B35-behavsci-16-01663] Flaspohler P., Duffy J., Wandersman A., Stillman L., Maras M. A. (2008). Unpacking prevention capacity: An intersection of research-to-practice models and community-centered models. American Journal of Community Psychology.

[B36-behavsci-16-01663] Flaspohler P. D., Meehan C., Maras M. A., Keller K. E. (2012). Ready, willing, and able: Developing a support system to promote implementation of school-based prevention programs. American Journal of Community Psychology.

[B37-behavsci-16-01663] Forman S. G., Olin S. S., Hoagwood K. E., Crowe M., Saka N. (2009). Evidence-based interventions in schools: Developers’ views of implementation barriers and facilitators. School Mental Health.

[B38-behavsci-16-01663] Goh A. E., Bambara L. M. (2012). Individualized positive behavior support in school settings: A meta-analysis. Remedial and Special Education.

[B39-behavsci-16-01663] Graham I. D., Logan J., Harrison M. B., Straus S. E., Tetroe J., Caswell W., Robinson N. (2006). Lost in knowledge translation: Time for a map?. Journal of Continuing Education in the Health Professions.

[B40-behavsci-16-01663] Grant S., Schweer-Collins M., Day E., Trevino S. D., Steinka-Fry K., Tanner-Smith E. E. (2025). Effectiveness of school-based depression prevention interventions: An overview of systematic reviews with meta-analyses on depression outcomes. Journal of Consulting and Clinical Psychology.

[B41-behavsci-16-01663] Green B. N., Johnson C. D., Adams A. (2006). Writing narrative literature reviews for peer-reviewed journals: Secrets of the trade. Journal of Chiropractic Medicine.

[B42-behavsci-16-01663] Green L. W. (2008). Making research relevant: If it is an evidence-based practice, where’s the practice-based evidence?. Family Practice.

[B43-behavsci-16-01663] Gregory A. T., Denniss A. R. (2018). An introduction to writing narrative and systematic reviews—Tasks, tips and traps for aspiring authors. Heart, Lung & Circulation.

[B44-behavsci-16-01663] Hanson R. F., Self-Brown S., Rostad W. L., Jackson M. C. (2016). The what, when, and why of implementation frameworks for evidence-based practices in child welfare and child mental health service systems. Child Abuse & Neglect.

[B45-behavsci-16-01663] Heatly M. C., Nichols-Hadeed C., Stiles A. A., Alpert-Gillis L. (2023). Implementation of a school mental health learning collaborative model to support cross-sector collaboration. School Mental Health.

[B46-behavsci-16-01663] Herlitz L., MacIntyre H., Osborn T., Bonell C. (2020). The sustainability of public health interventions in schools: A systematic review. Implementation Science.

[B47-behavsci-16-01663] Hoffmann J. A., Alegría M., Alvarez K., Anosike A., Shah P. P., Simon K. M., Lee L. K. (2022). Disparities in pediatric mental and behavioral health conditions. Pediatrics.

[B48-behavsci-16-01663] Hoover S., Bostic J. (2021). Schools as a vital component of the child and adolescent mental health system. Psychiatric Services.

[B49-behavsci-16-01663] Hoover S., Scardamalia K., Reaves S., Charlot-Swilley D., Trainor K., Morgan O., Bostic J. (2023). Organizational well-being inventory for schools (OWBI-S).

[B50-behavsci-16-01663] Institute of Medicine (1994). Reducing risks for mental disorders: Frontiers for preventive intervention research.

[B51-behavsci-16-01663] Khan S., Mnalili K., Moore J. E. (2024). White paper: Core competencies and functions of implementation support practitioners.

[B52-behavsci-16-01663] Konstantinidou I., Dillenburger K., Ramey D. (2023). Positive behaviour support: A systematic literature review of the effect of staff training and organisational behaviour management. International Journal of Developmental Disabilities.

[B53-behavsci-16-01663] Krach S. K., McCreery M. P., Monk M. M., Bagneris J. R. (2025). Fidelity in school-based positive behavioral interventions and supports: Current status of compliance. Journal of School Health.

[B54-behavsci-16-01663] Langley A. K., Nadeem E., Kataoka S. H., Stein B. D., Jaycox L. H. (2010). Evidence-based mental health programs in schools: Barriers and facilitators of successful implementation. School Mental Health.

[B55-behavsci-16-01663] Lee D. C., O’Brien K. M., Presseau J., Yoong S., McCrabb S., McDiarmid K., Lecathelinais C., Wolfenden L., Hodder R. K. (2026). Identifying effective behavior change techniques in interventions for enhancing the implementation of school-based policies and/or practices to prevent chronic disease in students: A secondary analysis of a systematic review. Translational Behavioral Medicine.

[B56-behavsci-16-01663] Leeb R. T., Danielson M. L., Claussen A. H., Robinson L. R., Lebrun-Harris L. A., Ghandour R., Bitsko R. H., Katz S. M., Kaminski J. W., Brown J. (2024). Trends in mental, behavioral, and developmental disorders among children and adolescents in the US, 2016–2021. Preventing Chronic Disease.

[B57-behavsci-16-01663] Lewis-Palmer T., Barrett S., Lewis (2004). Coaches self-assessment.

[B58-behavsci-16-01663] Litvitskiy N. S., Resnick M., Hall M., Flaspohler P. D. (2026). Core features of high school prevention programming: A systemized literature review. Oral presentation at the 13th Annual Southeastern School Behavioral Health Conference.

[B59-behavsci-16-01663] Lyon A. R., Bruns E. J. (2019). From evidence to impact: Joining our best school mental health practices with our best implementation strategies. School Mental Health.

[B60-behavsci-16-01663] Manning A. R. (2009). Bridging the gap from availability to accessibility: Providing health and mental health services in schools. Journal of Evidence-Based Social Work.

[B61-behavsci-16-01663] March A., Stapley E., Hayes D., Town R., Deighton J. (2022). Barriers and facilitators to sustaining school-based mental health and wellbeing interventions: A systematic review. International Journal of Environmental Research and Public Health.

[B62-behavsci-16-01663] McCrone P. (2025). Debate: Where to next for universal school-based mental health interventions? Universal versus targeted school-based mental health interventions: A health economic perspective. Child and Adolescent Mental Health.

[B63-behavsci-16-01663] McIntosh K., Massar M. M., Algozzine R. F., George H. P., Horner R. H., Lewis T. J., Swain-Bradway J. (2017). Technical adequacy of the SWPBIS tiered fidelity inventory. Journal of Positive Behavior Interventions.

[B64-behavsci-16-01663] McIsaac J. L. D., Penney T. L., Storey K. E., Sigfridson L., Cunningham J., Kuhle S., Kirk S. F. (2018). Integrated knowledge translation in population health intervention research: A case study of implementation and outcomes from a school-based project. Health Research Policy and Systems.

[B65-behavsci-16-01663] Melde C., Esbensen F. A., Tusinski K. (2006). Addressing program fidelity using onsite observations and program provider descriptions of program delivery. Evaluation Review.

[B66-behavsci-16-01663] Merle J. L., Thayer A. J., Larson M. F., Pauling S., Cook C. R., Rios J. A., McGinnis J. L., Sullivan M. M. (2022). Investigating strategies to increase general education teachers’ adherence to evidence-based social-emotional behavior practices: A meta-analysis of the single-case literature. Journal of School Psychology.

[B67-behavsci-16-01663] Merrill K. G., Dougherty A., Battalio S. L., Hartstein M. L., Silva A., Moskowitz D. A., De Pablo M. G., Margellos-Anast H., Baumann A. A., Navalpakkam P., Sandoval A., Bailey L., Chapman M., DiCesare J., Ortiz E., Habibi B., Bautista B. A., Martinez I., Wilson N., Martin M. A. (2025). Implementing capacity-building initiatives addressing health equity through community–academic partnerships: A qualitative study. Translational Behavioral Medicine.

[B68-behavsci-16-01663] Midwest PBIS (n.d.). Midwest PBIS network: Positive behavioral interventions and supports.

[B69-behavsci-16-01663] Moir T. (2018). Why is implementation science important for intervention design and evaluation within educational settings?. Frontiers in Education.

[B70-behavsci-16-01663] Mychailyszyn M. P., Brodman D. M., Read K. L., Kendall P. C. (2012). Cognitive-behavioral school-based interventions for anxious and depressed youth: A meta-analysis of outcomes. Clinical Psychology: Science and Practice.

[B71-behavsci-16-01663] Newton J. S., Algozzine B., Algozzine K., Horner R. H., Todd A. W. (2011). Building local capacity for training and coaching data-based problem solving with Positive Behavior Intervention and Support teams. Journal of Applied School Psychology.

[B72-behavsci-16-01663] Oakes W. P., Lane K. L., Germer K. A. (2014). Developing the capacity to implement tier 2 and tier 3 supports: How do we support our faculty and staff in preparing for sustainability?. Preventing School Failure: Alternative Education for Children and Youth.

[B73-behavsci-16-01663] Oddo L. E., McLeod B. D., Sutherland K. S., Chow J. C., Ledford J. R., Li G. W. (2025). A novel approach to research synthesis with the distillation and matching model: Application to the prevention of youth social, emotional, and behavioral problems. Prevention Science.

[B74-behavsci-16-01663] Osher D., Kidron Y., Brackett M., Dymnicki A., Jones S., Weissberg R. P. (2016). Advancing the science and practice of social and emotional learning: Looking back and moving forward. Review of Research in Education.

[B75-behavsci-16-01663] Owens J. S., Lyon A. R., Brandt N. E., Warner C. M., Nadeem E., Spiel C., Wagner M. (2014). Implementation science in school mental health: Key constructs in a developing research agenda. School Mental Health.

[B76-behavsci-16-01663] Pearrow M. M., Amador A., Dennery K. M. (2021). Community partnership consultation model: A consultative approach for supporting school-based behavioral health. Journal of Educational and Psychological Consultation.

[B77-behavsci-16-01663] Perkins K. A., Figas K., Westbrook A., Barrett S., Yanek K., Weist M. D. (2026). Development and evaluation of a university-based external coaching program for school behavioral health. Behavioral Sciences.

[B78-behavsci-16-01663] Pierce J. D. (2025). Translating systems coaching research to practice. Teaching Exceptional Children.

[B79-behavsci-16-01663] Price O. A., Long M., Sadlon R., Sheriff L. (2024). Enhancing school capacity to adopt school behavioural health best practices: A multiple-case study evaluation. Journal of Community & Applied Social Psychology.

[B80-behavsci-16-01663] Rapport F., Clay-Williams R., Churruca K., Shih P., Hogden A., Braithwaite J. (2018). The struggle of translating science into action: Foundational concepts of implementation science. Journal of Evaluation in Clinical Practice.

[B81-behavsci-16-01663] Richter A., Sjunnestrand M., Romare Strandh M., Hasson H. (2022). Implementing school-based mental health services: A scoping review of the literature summarizing the factors that affect implementation. International Journal of Environmental Research and Public Health.

[B82-behavsci-16-01663] Salimi Y., Shahandeh K., Malekafzali H., Loori N., Kheiltash A., Jamshidi E., Frouzan A. S., Majdzadeh R. (2012). Is community-based participatory research (CBPR) useful? A systematic review on papers in a decade. International Journal of Preventive Medicine.

[B83-behavsci-16-01663] Saunders M. N. K., Rojon C. (2011). On the attributes of a critical literature review. Coaching: An International Journal of Theory, Research and Practice.

[B84-behavsci-16-01663] Scaccia J. P., Cook B. S., Lamont A., Wandersman A., Castellow J., Katz J., Beidas R. S. (2015). A practical implementation science heuristic for organizational readiness: R = MC2. Journal of Community Psychology.

[B85-behavsci-16-01663] Schäfer S. K., Streit S., Schäfer C. G., Roembell L. B., Corneli M., Schaubruch L. M., Wessa M., Lieb K., Equit M., Fuhr D., STRESS-Care Consortium (2025). Barriers and facilitators for the implementation of preventative mental health interventions among secondary schools in high-income countries: A systematic review. European Child & Adolescent Psychiatry.

[B86-behavsci-16-01663] Schmittdiel J. A., Grumbach K., Selby J. V. (2010). System-based participatory research in health care: An approach for sustainable translational research and quality improvement. The Annals of Family Medicine.

[B87-behavsci-16-01663] Sklad M., Diekstra R., Ritter M. D., Ben J., Gravesteijn C. (2012). Effectiveness of school-based universal social, emotional, and behavioral programs: Do they enhance students’ development in the area of skill, behavior, and adjustment?. Psychology in the Schools.

[B88-behavsci-16-01663] Solomon B. G., Klein S. A., Politylo B. C. (2012). The effect of performance feedback on teachers’ treatment integrity: A meta-analysis of the single-case literature. School Psychology Review.

[B89-behavsci-16-01663] South Carolina School Behavioral Health Academy (2024). The South Carolina school behavioral health academy seeks to help districts respond to the youth mental health crisis by integrating behavioral health into the multi-tiered system of supports (MTSS).

[B90-behavsci-16-01663] Splett J. W., Perales K., Miller E., Hartley S. N., Wandersman A., Halliday C. A., Weist M. D. (2022). Using readiness to understand implementation challenges in school mental health research. Journal of Community Psychology.

[B91-behavsci-16-01663] Stanford Medicine (n.d.). 508 compliant implementation guidance modules.

[B92-behavsci-16-01663] Stormont M., Reinke W. M., Newcomer L., Marchese D., Lewis C. (2015). Coaching teachers’ use of social behavior interventions to improve children’s outcomes: A review of the literature. Journal of Positive Behavior Interventions.

[B93-behavsci-16-01663] Sugai G., Horner R. R. (2006). A promising approach for expanding and sustaining school-wide positive behavior support. School Psychology Review.

[B94-behavsci-16-01663] Sugai G., LaMaster D. (2015). PBIS forum 15 practice brief: District level coaching.

[B95-behavsci-16-01663] Sukhera J. (2022). Narrative reviews: Flexible, rigorous, and practical. Journal of Graduate Medical Education.

[B96-behavsci-16-01663] Taylor R. D., Oberle E., Durlak J. A., Weissberg R. P. (2017). Promoting positive youth development through school-based social and emotional learning interventions: A meta-analysis of follow-up effects. Child Development.

[B97-behavsci-16-01663] University of Maryland (2026). National center for school mental health (NCSMH).

[B98-behavsci-16-01663] Wandersman A., Cook B. S., Clark K., Flaspohler P., Watson A., Lamont A. E. (2024). Commentary: Bridging and reducing the gaps between research and practice: Pathways to outcomes and the interactive systems framework for dissemination and implementation 2.0. Evaluation & The Health Professions.

[B99-behavsci-16-01663] Wandersman A., Duffy J., Flaspohler P., Noonan R., Lubell K., Stillman L., Blachman M., Dunville R., Saul J. (2008). Bridging the gap between prevention research and practice: The interactive systems framework for dissemination and implementation. American Journal of Community Psychology.

[B100-behavsci-16-01663] Wandersman A., Imm P., Chinman M., Kaftarian S. (2000). Getting to outcomes: A results-based approach to accountability. Evaluation and Program Planning.

[B101-behavsci-16-01663] Wandersman A., Scheier L. M. (2024). Strengthening the science and practice of implementation support: Evaluating the effectiveness of training and technical assistance centers. Evaluation & The Health Professions.

[B102-behavsci-16-01663] Ward C. S., Farmer S., Livet M. (2024). Technical assistance for systemic change: Lessons learned from a national technical assistance center. Evaluation & The Health Professions.

[B103-behavsci-16-01663] Weist M. D., Figas K., Stern K., Terry J., Scherder E., Collins D., Davis T., Stevens R. (2022a). Advancing school behavioral health at multiple levels of scale. Pediatric Clinics of North America.

[B104-behavsci-16-01663] Weist M. D., Franke K. B., Stevens R. N. (2020). School behavioral health: Interconnecting comprehensive school mental health and positive behavior support.

[B105-behavsci-16-01663] Weist M. D., Splett J. W., Halliday C., Seaman M. A., Gage N., Perkins K., Perales K., Miller E., McCollister K., Collins D., Rizzardi V., DiStefano C. (2021). Interconnecting PBIS and school mental health to improve school safety: A randomized trial.

[B106-behavsci-16-01663] Weist M. D., Splett J. W., Halliday C. A., Gage N. A., Seaman M. A., Perkins K. A., Perales K., Miller E., Collins D., DiStefano C. (2022b). A randomized controlled trial on the interconnected systems framework for school mental health and PBIS: Focus on proximal variables and school discipline. Journal of School Psychology.

[B107-behavsci-16-01663] Westerlund A., Nilsen P., Sundberg L. (2019). Implementation of implementation science knowledge: The research-practice gap paradox. Worldviews on Evidence-Based Nursing.

[B108-behavsci-16-01663] Wolk C. B., Stewart R. E., Eiraldi R., Cronholm P., Salas E., Mandell D. S. (2019). The implementation of a team training intervention for school mental health: Lessons learned. Psychotherapy.

[B109-behavsci-16-01663] Woolf S. H. (2025). The youth mental health crisis in the United States: Epidemiology, contributors, and potential solutions. Pediatrics.

