# Supplementary material for: Advancing School Behavioral Health Implementation: A Critical Review Using ISF 2.0
Source: Behav Sci (Basel). 2026 Sep 16;16(9):1663. doi: 10.3390/bs16091663 (PMC13603748; doi:10.3390/bs16091663)
Supplement: Supplementary file 1 [file behavsci-16-01663-s001.zip › behavsci-4440993-supplementary.pdf]

Supplementary File S1

Table S1

Case Examples Mapped Across ISF 2.0 Systems

System	NCSMH	Center on PBIS	SBHA	Summary of Analysis
Synthesis & Translation	SHAPE: quality guides, assessments, implementation guidance, policy resources	PBIS blueprints, readiness and implementation assessments, practice guides, tools, webinars	LMS: 43+ hours of interactive asynchronous SBH training and resources	Evidence is translated into accessible resources, tools, training, and implementation guidance designed primarily for delivery system providers, as well as some implementation support providers. Some mechanisms exist for incorporating practice-based feedback to inform and improve products. Products skew toward addressing innovation-specific capacity. There are opportunities to more intentionally align synthesis and translation efforts with implementation support and develop resources that address motivation, general capacity, and implementation processes.
Support	Training and technical assistance, learning collaboratives, webinars, coaching, leadership training, quality improvement support	Cascade model prescribing regional, state, and district coaching infrastructure; Midwest PBIS training, resources, and fee-based coaching services	Freely accessible tailored systems coaching supporting teaming, leadership, planning, data use, quality improvement, and coaching capacity building	The existing continuum of support includes training, technical assistance, coaching, and learning collaboratives. Implementation support infrastructure, accessibility of support, and the targets of support vary. Opportunities remain to further formalize support models, strengthen evaluation of support activities, and expand internal support infrastructure.

Delivery	National conference, direct school support, practitioner listservs, research/evaluation partnerships	Multilevel PBIS teams; annual conferences promoting horizontal and vertical exchange of practice-based knowledge	Direct support to districts and schools; engagement through webinars and conference	Delivery systems are engaged through partnerships with schools, districts, and state agencies. Some mechanisms exist for building partnerships across delivery systems and for delivery system providers to provide feedback to other systems. Existing initiatives emphasize innovation-specific capacity. There are opportunities to strengthen systematic feedback processes and recalibrate efforts to enhance general capacity and motivation alongside innovation-specific capacity.
Cross-System interaction	Strong integration of research, synthesis/translation, support, and evaluation within selected initiatives; less explicit routine upstream feedback	Strong multilevel dissemination; opportunities for bidirectional exchange through conferences; limited or unclear systematic feedback mechanisms	Explicit bidirectional feedback between LMS, coaching, and delivery teams; feedback informs revisions although feedback mechanisms are not robust	Bidirectional connections and feedback mechanisms across systems are often underdeveloped or unclear, particularly in upstream direction.
Opportunities for Enhanced Alignment	Expand explicit connections between synthesis/translation and support; strengthen upstream feedback mechanisms; broaden readiness focus	Link resources more directly to coaching; strengthen local support and feedback; broaden readiness focus	Systematize feedback to aggregate feedback across coaching and LMS activities; expand communities of practice to strengthen cross-site learning; better align training and coaching around readiness	Strengthen communication channels between systems through explicit methods. Create systematic process for collecting and integrating feedback across systems. Ensure all components of readiness are addressed within each system.
